# Development of gut microbiota along with its metabolites of preschool children

**DOI:** 10.1186/s12887-021-03099-9

**Published:** 2022-01-06

**Authors:** Jingjing Xiong, Hongwei Hu, Chuanzhi Xu, Jianwen Yin, Mei Liu, Lizhi Zhang, Yong Duan, Yongkun Huang

**Affiliations:** 1 Department of Pediatrics, the First Affiliated Hospital of Kunming Medical University, No. 295, Xichang Road, Xishan District, Kunming, 650032 China; 2 Department of Statistics, School of Public Health, Kunming Medical University, Kunming, 650000 China; 3 Yunnan Center for Disease Control and Prevention, 158 Dongsi Street, Xishan District, Kunming, 650022 Yunnan China; 4 Department of Clinical Laboratory, the First Affiliated Hospital of Kunming Medical University, Kunming, 650032 China

**Keywords:** Gut microbiota, Children development, Microbial metabolism, Bile acids, Short chain fatty acids

## Abstract

**Background:**

To reveal the changes of intestinal microbial abundance and composition, as well as the microbiota metabolic levels of bile acids and short chain fatty acids of healthy preschool children during their growth.

**Methods:**

Feces of 120 healthy newborns and 150 healthy children aged 6 months to 6 years were collected. Then the composition of intestinal flora was analyzed by 16S rRNA, and the contents of bile acids and short chain fatty acids in feces were detected by LC-MS and GS methods, respectively.

**Results:**

The composition and function of intestinal microflora were not stable in neonatal period but significantly improved at 6 months after birth, and gradually stabilized and tended to adult-like formation after 2–3 years old. The levels of short chain fatty acids and secondary bile acids were consistent with the development of gut microbiota.

**Conclusion:**

The age of 6 months may be a critical period for the development of intestinal microflora in children.

**Supplementary Information:**

The online version contains supplementary material available at 10.1186/s12887-021-03099-9.

## Background

Gastrointestinal microorganisms are a diverse community of bacteria, archaea, fungi, unicellular organisms, and viruses, which inhabit the intestines of all mammals. Studies on humans and other mammals have shown that gut microbes are involved in a series of physiological processes that are critical to host health, including energy homeostasis, metabolism, intestinal epithelial health, immune activity, and neurobehavioral development [1, 2]. Changes in gastrointestinal microbiota are reported to associate with the occurrences and developments of multisystem diseases, including inflammatory bowel disease, asthma, obesity, metabolic syndrome, cardiovascular disease, immune system disease and neurodevelopmental conditions, such as autism spectrum disorders [3–6]. The interaction between the gut microbes and the host has a profound impact on the development of health and diseases [7].

Human gut microbiota plays an important role in health and disease from prenatal to childhood. Studies have shown that the microorganisms in the human body at the early stage of birth play an important role in the regulation of human development and maturity [8]. A study on human microbiota in the first 1000 days (from pregnancy to 2 years old) showed that intestinal microbial succession has an important impact on the growth and development of children [9]. There are studies on the gut microecology of preterm infants and the influence of feeding methods on the gut microflora development of preterm infants [10], the influence of delivery mode on the composition of gut microflora [11], and the gut microflora of children with autism spectrum disorder [12, 13]. From infant to pre-school age (0–6 years old) is a critical period for the growth and development of children’s various systems, but the development and succession of gut microflora of healthy 0–6-year-old children remains poorly revealed.

The gastrointestinal microorganism is considered as a metabolic “organ” and central regulator of host metabolism which can not only help decompose and absorb of nutrients and energy from food but also produce numerous metabolites and regulate host metabolism [14]. Recently, with the further research of gastrointestinal microorganism, the short chain fatty acids (SCFAs) and bile acids (BAs) are two of the major metabolites produced by gut microbiota and paly important role in human homeostasis and health [15, 16]. Dietary fibers are metabolized into SCFAs, mainly acetate, propionate, and butyrate by the fermentation of intestinal microorganisms [17]. In this process, the host biosynthesis of primary bile acids (PBAs), which then enters the intestine through the liver-gut axis and is uncoupled and transformed into secondary bile acids (SBAs) by intestinal microbiota [18], and that’s why the bile acid metabolism is also a typical example of host-gut microbial co-metabolism. From the beginning of life, the composition and function of intestinal microflora are dynamic and mature with age. The composition and development of intestinal microflora are affected by diet structure, environment, and other factors. At the same time, it affects host health and disease occurrence and development by regulating the metabolism level of the host.

In order to reveal the composition and function changes of gut flora in preschool children from birth to 6 years old, we analyzed and compared the gut flora composition of 120 newborns born in the First Affiliated Hospital of Kunming Medical University in 2015 and 150 healthy children aged from 6 months to 6 years old who underwent physical examination in the First Affiliated Hospital of Kunming Medical University from 2015 to 2016 in the present study. Meanwhile the levels of BAs and SCFAs in the feces of these participants, which can reflect the influence of gut flora on metabolic level in a certain extent, were also determined.

## Methods

### Participants and sample collection

All human studies were approved by the Hospital Ethical Committee of First Affiliated Hospital of Kunming Medical University in Yunnan Province, China, and written informed consents were obtained from the custodians of each participant before samples collecting. A total of 120 neonates (0–28 days after birth) all came from obstetrics department of the First Affiliated Hospital of Kunming Medical University in 2015. All the newborns were born in term labor with an average weight of 3.21 ± 0.23 kg, breastfed after birth, mothers were in good health during pregnancy and did not use antibiotics before delivery. One hundred fifty healthy children aged from 6 months to 6 years old who underwent physical examination in the First Affiliated Hospital of Kunming Medical University from 2015 to 2016 were selected and met the following criteria: a) body mass index was in the normal range, and the dietary structure of each age group was similar; b) no allergic disease, gastrointestinal disease, or immune system disease; no family history of genetic and metabolic diseases; c) no antibiotics and probiotics were used in the past 4 weeks. These 270 participants were divided into nine groups (*n* = 30 per group) according to the ages: 3 d-old group (g group), 7 d-old group (h group), 14 d-old group (i group), 25 d-old group (j group), 6 m-old group (f group), 1 y-old group (e group), 2 y-old group (d group), 3 y-old group (c group) and 6 y-old group (b group).

Stool samples were collected from participants of each group, kept at − 20 °C upon defecation and within the same day delivered frozen to Main Microbiology laboratory of the First Affiliated Hospital of Kunming Medical University, where aliquots of each sample were stored at − 80 °C for further processing. The study design overview was shown as Fig. S1.

### Gut microbiome measurement

#### DNA extractions

DNA from different samples was extracted using the E.Z.N.A.®Stool DNA Kit (D4015, Omega, Inc., USA) according to manufacturer’s instructions. The reagent which was designed to uncover DNA from trace amounts of sample has been shown to be effective for the preparation of DNA of most bacteria. Nuclear-free water was used for blank. The total DNA was eluted in 50 μL of Elution buffer and stored at − 80 °C until measurement in the PCR by LC-Bio Technology Co., Ltd., Hang Zhou, Zhejiang Province, China.

#### PCR amplification and 16S rDNA sequencing

The V3-V4 region of the prokaryotic (bacterial and archaeal) small-subunit (16S) rRNA gene was amplified with primers 341F (5′-CCTACGGGNGGCWGCAG-3′) and 785R (5′-GACTACHVGGGTATCTAATCC-3′) [19]. The 5′ ends of the primers were tagged with specific barcodes per sample and sequencing universal primers. PCR amplification was performed in a total volume of 25 μL reaction mixture containing 25 ng of template DNA, 12.5 μL PCR Premix, 2.5 μL of each primer, and PCR-grade water to adjust the volume. The PCR conditions to amplify the prokaryotic 16S fragments consisted of an initial denaturation at 98 °C for 30 s; 32 cycles of denaturation at 98 °C for 10 s, annealing at 54 °C for 30 s, and extension at 72 °C for 45 s; and then final extension at 72 °C for 10 min. The PCR products were confirmed with 2% agarose gel electrophoresis. Throughout the DNA extraction process, ultrapure water, instead of a sample solution, was used to exclude the possibility of false-positive PCR results as a negative control. The PCR products were purified by AMPure XT beads (Beckman Coulter Genomics, Danvers, MA, USA) and quantified by Qubit (Invitrogen, USA). The amplicon pools were prepared for sequencing and the size and quantity of the amplicon library were assessed on Agilent 2100 Bioanalyzer (Agilent, USA) and with the Library Quantification Kit for Illumina (Kapa Biosciences, Woburn, MA, USA), respectively. The libraries were sequenced on NovaSeq PE250 platform.

#### Data analysis

Samples were sequenced on an Illumina NovaSeq platform according to the manufacturer’s recommendations, provided by LC-Bio. Paired-end reads was assigned to samples based on their unique barcode and truncated by cutting off the barcode and primer sequence. Paired-end reads were merged using FLASH. Quality filtering on the raw reads were performed under specific filtering conditions to obtain the high-quality clean tags according to the fqtrim (v0.94). Chimeric sequences were filtered using Vsearch software (v2.3.4). After dereplication using DADA2,we obtained feature table and feature sequences. Alpha diversity and beta diversity were calculated by QIIME2,which the same number of sequences were extracted randomly through reducing the number of sequences to the minimum of some samples, and the relative abundance (X bacteria count/total count) is used in bacteria taxonomy. Alpha diversity and Beta diversity were analyzed by QIIME2 process, and pictures were drawn by R (v3.5.2). The sequence alignment of species annotation was performed by QIIME2 plugin feature-classifier, and the alignment database was SILVA and NT-16S.

### Determination of bile acid level

The concentration of bile acids in feces was analyzed by LC-MS. Before the test, 10 mg fecal samples were mixed with 50 μL of methanol internal standard solution containing cholic acid-D4, chenodeoxycholic acid-D4, lithocholic acid-D4, deoxycholic acid-D4, and ursodeoxycholic acid-D4 (Cambridge Isotope Laboratory), and incubated overnight at 4 °C with 6 ml of chloroform: methanol (2:1, volume: Volume) solution. Then PBAs and SBAs were quantified by LC-quadrupole time-of-flight/MS isotope dilution method according to the steps previously reported [20]. The data were standardized by the dry weight of fecal samples and expressed in pg/g.

### Determination of SCFAs level

The fecal samples were weighed and suspended in 1 mL water, containing 9% formic acid per 0.1 g, then homogenized by a vortex for 2 min, and centrifuged at 12000×g for 15 min. Each mL of water supernatant was extracted with 1 mL ether at 4 °C for 8 h, and then centrifuged at 12000×g for 10 min. Diethyl ether extracts was transferred into the sample bottle for further measure.

The quantitative determination of SCFA in fecal samples was carried out by gas chromatography equipped with flame ionization detector (FID) and N10149 automatic liquid sampler (Agilent, USA), and all operating procedures were carried out in accordance with previous reports [10].

### Statistical analysis

Depending on the data distribution, a Kruskal-Wallis test, or One-way ANOVA test followed by Bartlett’s test for equal variances (Bonferroni’s Multiple Comparison Test, 95% CI) was used to calculate significance levels between experimental groups. *P* < 0.05 was considered significant. Graph generation and statistical analyses were performed using GraphPad Prism 5.

## Results

### Diversities of gut Flora

**Fig. 1:**
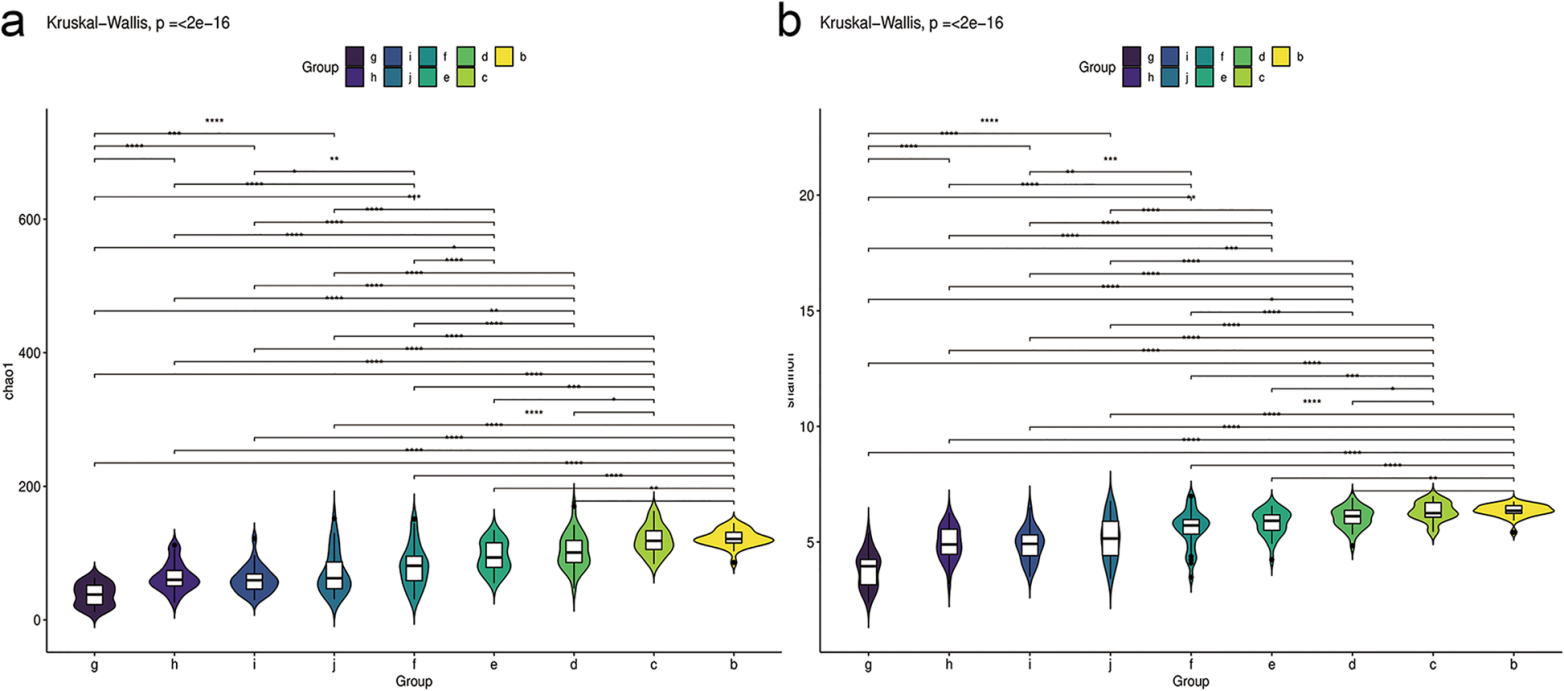
The alpha diversity of the gut flora in different groups. **a** Chao 1 index and **b** Shannon index indicated the richness and evenness of the species in each group, respectively. Kruskal-Wallis test was used to determine the difference among these 9 groups. *p < 0.05, **p < 0.01, ***p < 0.001

The intestinal microflora diversity of the 9 participant groups was evaluated by alpha diversity and beta diversity. As shown in Fig. 1, both the richness (Chao 1 index) and evenness (Shannon index) of the gut flora species kept in a lower level during neonate period (g - j groups) but obviously increased from 6-month-old (f group), then stabled and kept in a higher level from 3-year-old to 6-year-old (c – b groups).

**Fig. 2:**
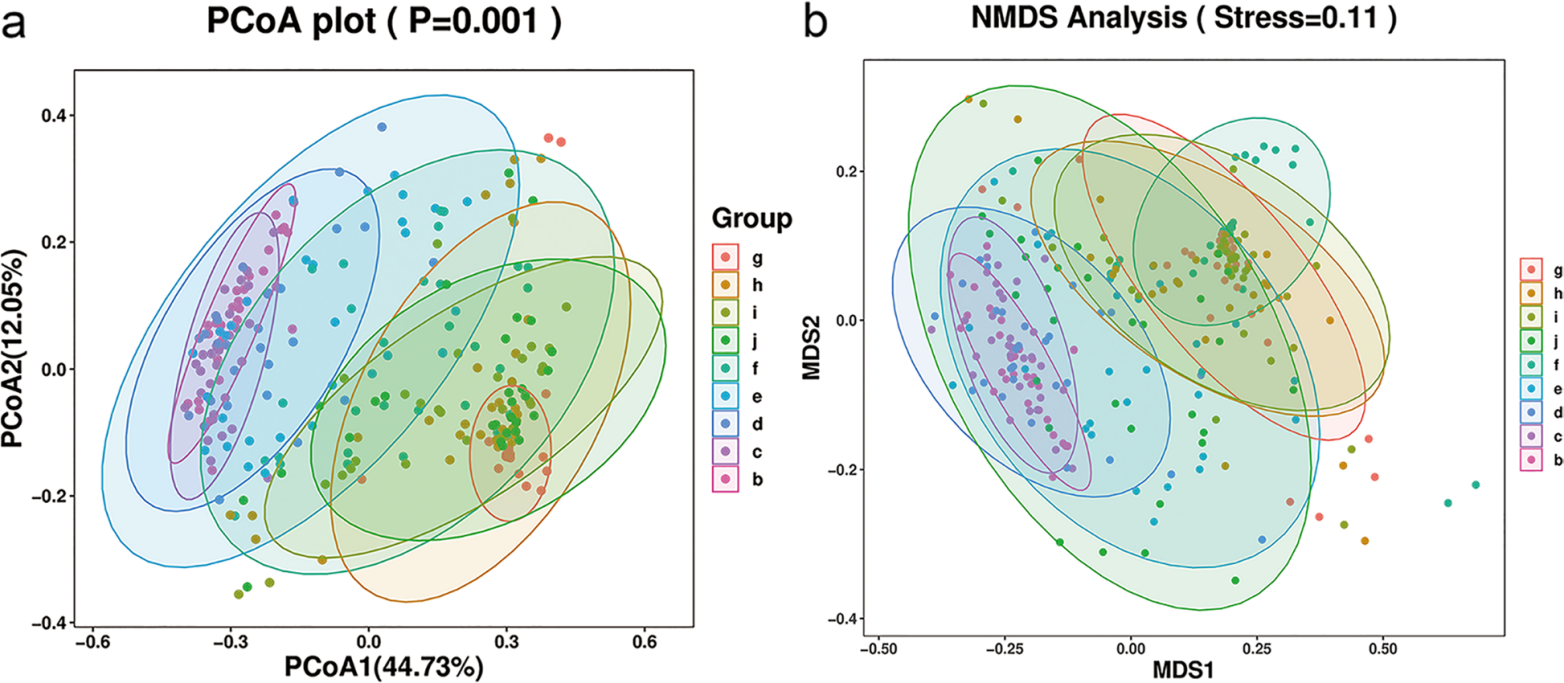
The beta diversity of the gut flora in different groups. **a** Principal coordinates analysis (PCoA) and **b** Multidimensional scaling (NMDS) were performed to detect the species diversity among different environmental communities. The weighted unifrac results were shown in the figure to establish the differences of beta diversity among these 9 groups

Beta diversity refers to the species diversity among different environmental communities. The weighted PCoA results showed the sample dispersion in 3-day-old group (g group) was in lowest level and began to increase from neonate period to 2-year-old (h – d groups), then decreased from 3-year-old (c group) and kept in a lower level of 6-year-old group (b group) (Fig. 2a). The NMDS analysis was also to detect the species similarity among samples. The results showed that the species compositions from birth to 14-day-old (g – I groups) were similar, and the distance between 3-year-old (c group) and 6-year-old (b group) was less than it between other groups (Fig. 2b).

### Compositions and functions of gut Flora

**Fig. 3:**
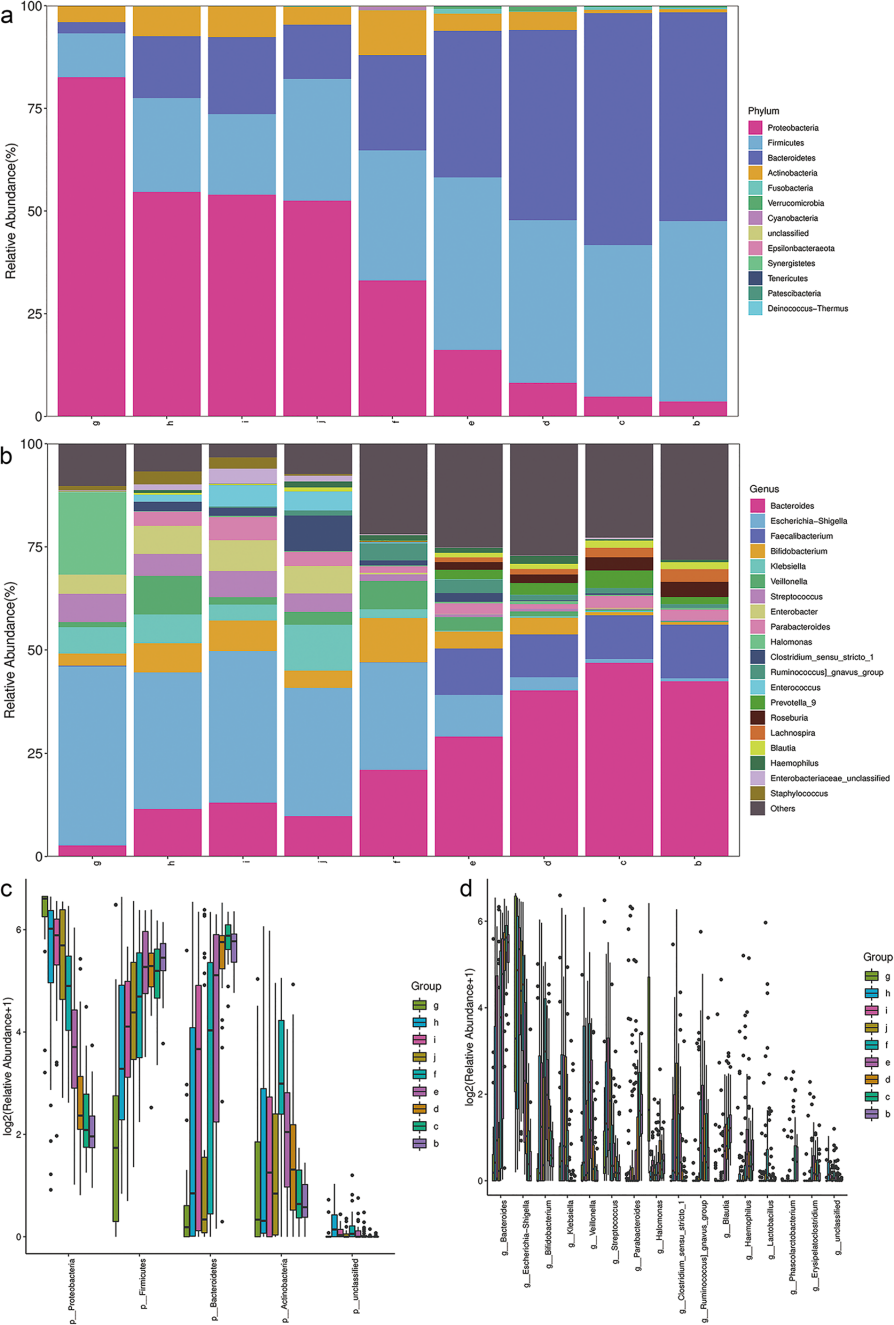
The species abundances of gut flora in different groups. **a** Phylum abundances and **b** Genus abundances were shown to establish the composition of species communities in each group. **c** and **b** showed the differences of abundances of main Phylum and Genus communities, respectively

To analyze the species composition of each sample, the SILVA (Release 132, *https://www.arb-silva.de/documentation/release-132/*) and NT-16S databases were used for species annotation and stacked bar chart was used to exhibit the species compositions in each group. As shown in Fig. 3a, there were four major phyla in the gut flora: *Proteobacteria*, *Firmicutes*, *Bacteroidetes*, *Actinobacteria*, the abundance of *Proteobacteria* decreased gradually from birth to 6 years old, in contrary with the increasing of the abundances of *Firmicutes* and *Bacteroidetes*. The relative abundances of phylum showed that *Proteobacteria* was the main composition of gut flora during the neonate period (g – j groups) while the dominant populations became *Firmicutes* and *Bacteroidetes* from 6-month-old to 6-year-old (f – b groups) (Fig. 3a and c). And the genus abundance showed that the gut flora in neonate was mainly composed by *Escherichia-Shigella* (g – j groups) while the abundance of *Bacteroides* and other species gradually increased to become the dominant population from 6-month-old to 6-year-old (f – b groups) (Fig. 3b and d).

**Fig. 4:**
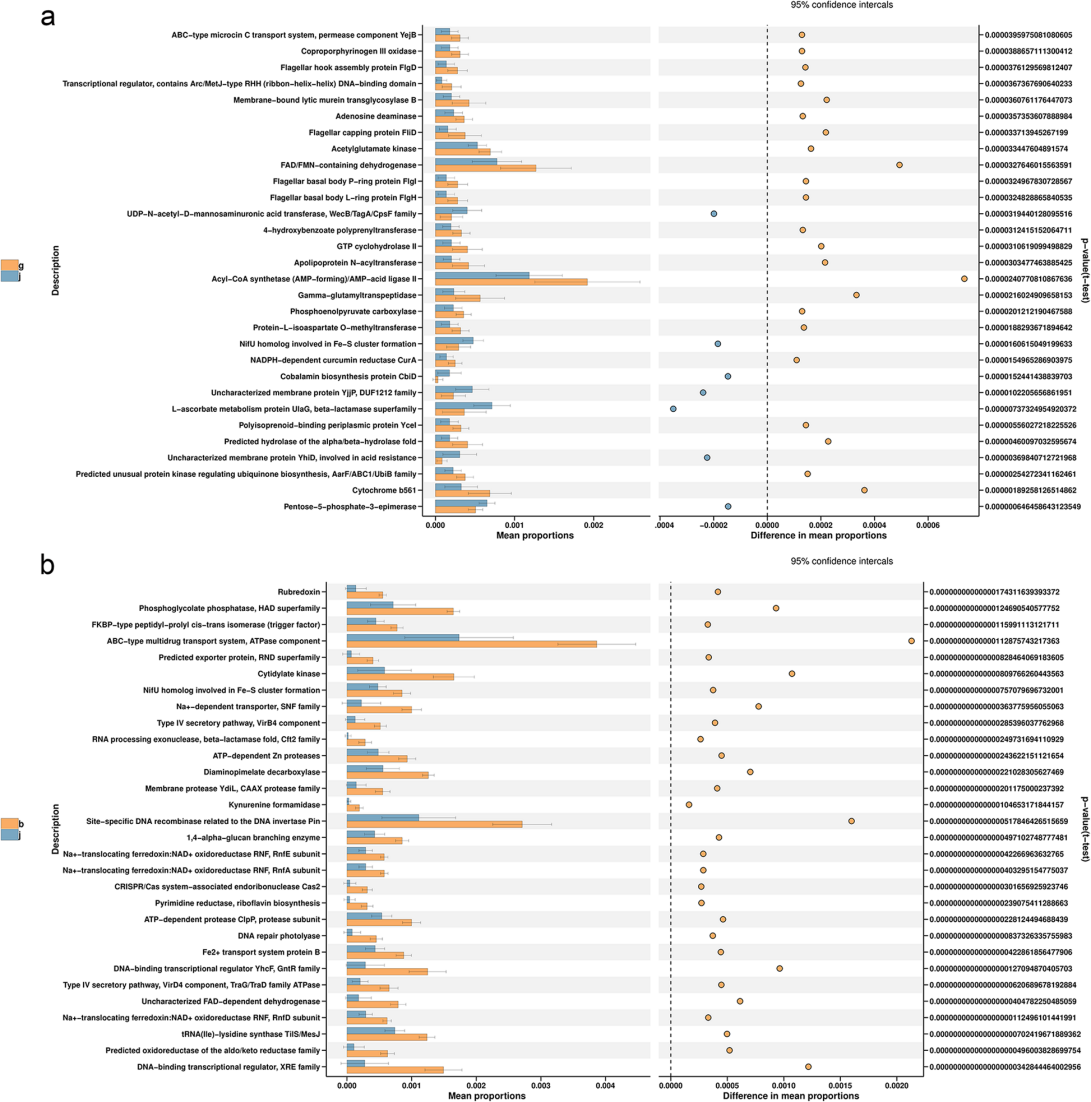
The functional prediction of intestinal flora. The functional differences between 3-day old (g group) and 25-day old (j group) (**a**), and between 25-day old (j group) and 6-year old (b group) (**b**) were predicted by PICRUSt2 method and the results from COG dataset were shown in the figure

To reveal the functional differences of gut flora among the samples, the function prediction was performed by PICRUSt2 (Phylogenetic Investigation of Communities by Reconstruction of Unobserved States, *https://github.com/picrust/picrust2*), and relative database of COG, EC, KO, PFAM, TIGRFAM were used to note the functions of the intestinal flora. Here just showed the results from COG dataset. The results showed that there was no significant functional difference between 3 d and 25 d groups, and the functions of gut flora in neonates were mainly enriched in FAD/FMN-containing dehydrogenase and AMP-forming/AMP-acid ligase II and others (Fig. 4a). On the contrary, the functions of gut flora in 25 d group and 6 y group showed obvious difference in ATPase component, Cytidylate kinase, Site-specific DNA recombinase related to the DNA invertase Pin, DNA-binding transcriptional regulator-XRE family, and other functions (Fig. 4b).

### Level of bile acids

**Fig. 5:**
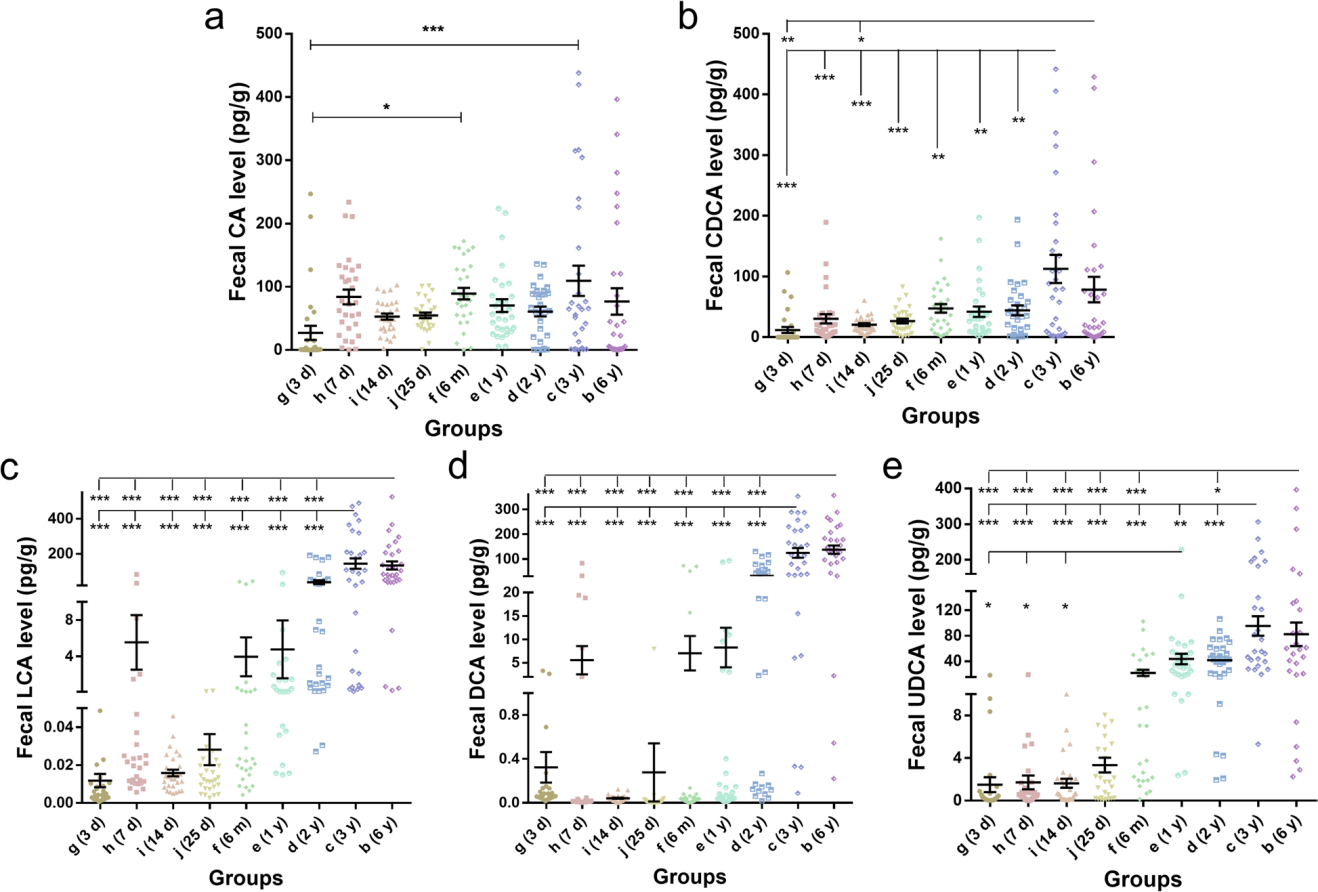
The fecal bile acids content in different groups. The fecal content of primary bile acids (PBAs) of cholic acid (**a**) and chenodeoxycholic acid (**b**) and secondary bile acids (SBAs) of lithocholic acid (**c**), deoxycholic acid (**d**) and ursodeoxycholic acid (**e**) were measured by LC-MS. A one-way ANOVA test was used to determine the differences among these 9 groups. *p < 0.05, **p < 0.01, ***p < 0.001

In the present study, we detected the concentrations of both primary bile acids (PBAs) and secondary bile acids (SBAs) in all 270 stool samples of nine groups of the participants. The results showed that there was no significant difference in fecal concentrations of PBAs, including cholic acid (CA) and chenodeoxycholic acid (CDCA), among the nine groups (Fig. 5a, b). As shown in Fig. 5a, the fecal CA content of newborn infants was extremely low only at 3 days after birth, and then it increased and seemed to remain stable until 6 years old. In addition, there were significant individual differences in fecal CA content among subjects in 3 d, 7 d, 1 y, 3 y, and 6 y groups, especially in 3 y group and 6 y group. Meanwhile, the fecal CDCA content was at a low level from 3 days to 2 years old, while the fecal CDCA level was rarely increased after 3 years old (Fig. 5b). However, there were individual differences of fecal CDCA contents in 3 y and 6 y old children, and the data of these two groups showed obvious dispersion. Different from the changes of the content of primary bile acids, the contents of SBAs, including lithocholic acid (LCA), deoxycholic acid (DCA) and ursodeoxycholic acid (UDCA) in the stool samples of 9 groups were significantly different (Fig. 5c-e); the level of fecal SBAs was lower in neonatal period (3d-25d), then began to increase from 6 m, and remained stable after 3 years old. It should be noted that the fecal LCA and DCA levels of the 7 d newborns had a significant sudden increasing, but the individual differences within the group were obviously observed, and the data dispersion was remarkably high (Fig. 5c, d). In general, the PBAs metabolism did not change significantly from birth to preschool, while the SBAs metabolism changed significantly around 6 m and tended to be stable after 3 years old.

### Level of short chain fatty acids (SCFAs)

**Fig. 6:**
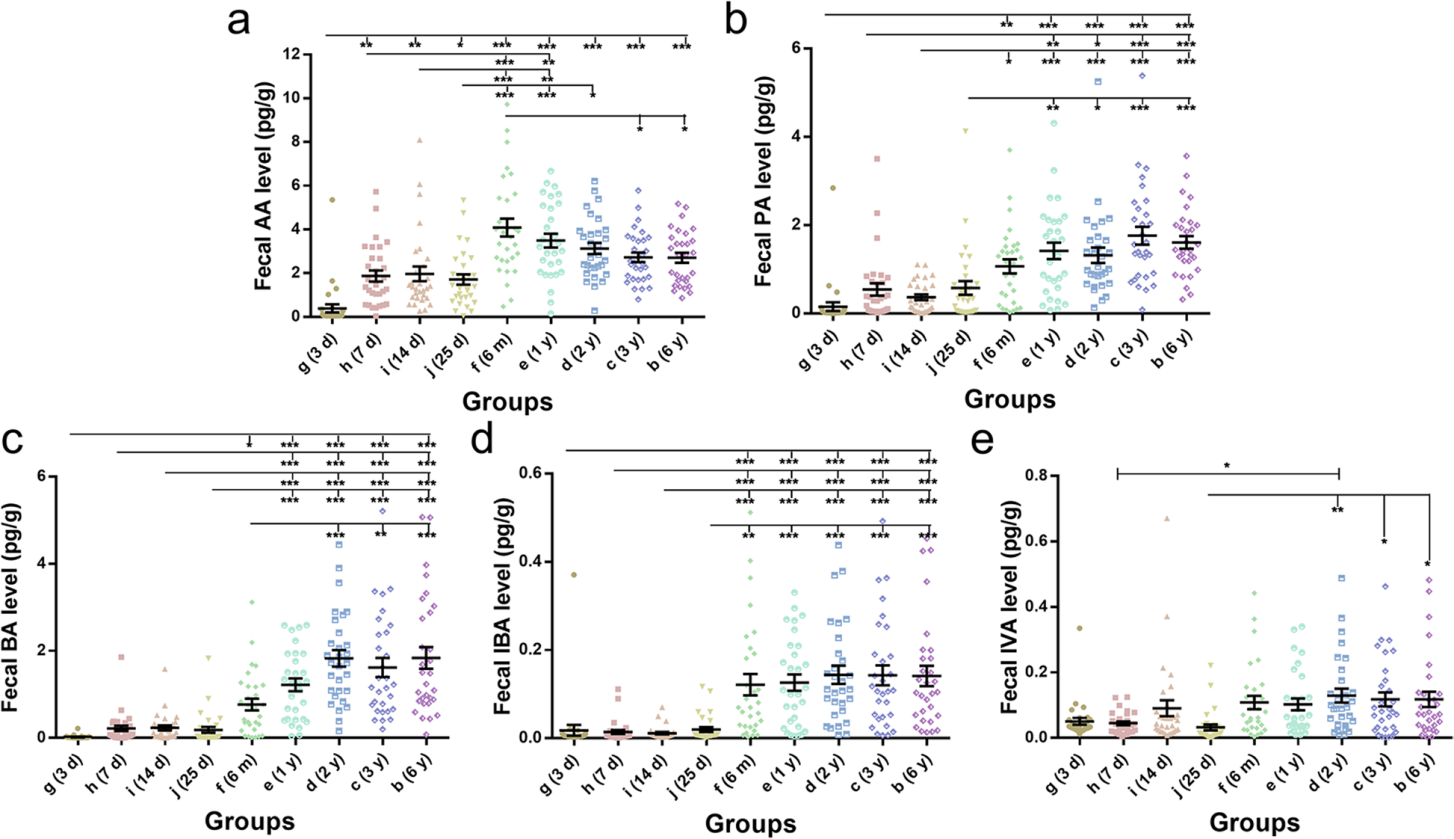
The fecal short chain fatty acids content in different groups. The fecal content of short chain fatty acids (SCFAs) of acetic acid (**a**), propionate (**b**), butyrate (**c**), isobutyric acid (**d**), and isovaleric acid (**e**) were measured by GS. A one-way ANOVA test was used to determine the differences among these 9 groups. *p < 0.05, **p < 0.01, ***p < 0.001

The study examined the concentrations of acetic acid (AA), propionate (PA), butyrate (BA), isobutyric acid (IBA), and isovaleric acid (IVA) in all 270 stool samples of nine groups of the participants. It can be seen from the results that there were obvious individual differences of fecal AA contents in each group, but overall, the fecal AA level was lower in the 3 d neonates, and there was no significant difference from 7 d to 25 d, but with a certain increasing compared with 3 d, then the AA level increased significantly at 6 m with highest data dispersion in this group (Fig. 6a). Thereafter, the fecal AA level gradually stabilized until 6 y and remained at a higher level than neonatal period accompanying the reducing of individual difference. The changes of PA and BA were similar, both of them kept a lower level in the neonatal period (from 3 d to 25 d), and gradually increased with age from 6 m to 6 y and tended to stable after 2 years old (Fig. 6b, c). As Fig. 6d shown, the level of IBA did not change significantly from 3 d to 25 d, and remained at a very low level; then it increased significantly at 6 m after birth, and remained unchanged after that, but with obvious individual difference. The concentration of fecal IVA was at low lever, and it seemed that there was an up regulation of IVA at 6 m (without statistical significance); after that, the average content of IVA remained at this level but lager individual difference (Fig. 6e). In summary, the levels of the SCFAs kept in lower levels in neonatal period and showed an obvious increasing at 6 m after birth except UDCA, and all of them tended to be stable after 2 years old.

## Discussion

In the past few years, more and more reports have revealed that there is a critical relationship between gut microflora and various chronic disease. The intestinal microecology acted on human health through gut-liver axis, gut-lung axis and gut-brain axis and so on [21]. Intestinal microbes play an important role in mammalian homeostasis and health [22], including providing essential nutrients [23], metabolizing dietary fiber into short chain fatty acids (SCFAs) [24], and ensuring the normal development of the immune system [25]. Therefore, intestinal microbiota was considered to be a key factor affecting early life development and lifelong health.

As previous studies reported, the complete colonization of gut microbiota may occurred at prior to birth [26], and the gut microbiota developed into a relatively stable, adult-like configuration within the first 3 years of life [27]. The results showed that from birth to 6 m after birth, the formation of intestinal microflora in infants was mainly related to delivery mode, feeding mode, antibiotic exposure and environmental factors; then mainly affected by weaning and solid food intake from 6 m to 18 m; and gradually tended to be a relatively stable and adult-like formation from 18 m to 36 m [11]. In addition, some studies have demonstrated that the development of pediatric intestinal flora lasts from childhood to adolescence [28, 29]. Ringel-Kulka T et al. [29] has reported that the diversity of intestinal microflora (both abundance and richness) in adults is significantly greater than that in 1–4 years old children, and there are differences in multiple phylogenetic groups between children and adults at the genus level.

The intestinal microflora diversity was evaluated by alpha diversity and beta diversity in our research. As we all know, alpha diversity refers to the diversity within a specific environment or ecosystem, mainly used to reflect species richness and evenness and sequencing depth and alpha diversity is mainly through the indexes of Chao1, Observed species, Goods coverage, Shannon and Simpson were used to reflect the richness and uniformity of the species in samples. Beta diversity refers to the species diversity among different environmental communities. Beta diversity and alpha diversity together constitute the overall diversity or the biological heterogeneity of certain environmental communities. The aim of PCoA analysis is to observe the difference between individual samples or groups of samples, the distance between different samples represents the difference of species composition. Our results showed that the Chao 1 index which indicated the abundance of gut flora was lower at neonatal period and obviously increased at 6 m while the Shannon index which indicated the uniformity of gut flora was gradually increased from birth to 6 years old (Fig. 1). After 3 years old, the intestinal microbial community of children has shown a similar composition to that of adults, and is mainly characterized by two major phyla: *Bacteroidetes* and *Firmicutes* (Fig. 3). These results are consistent with previous reports and suggested that it may be a critical period for the development of intestinal flora in infants around 6 months of birth.

In addition to intestinal microflora, a large number of intestinal microbial metabolites are also one of the components of intestinal microecology. SCFAs (mainly acetate, butyrate, and propionate) produced by intestinal microbial ferment dietary fiber are important functional substances in intestinal microenvironment, which are produced by intestinal microbial ferment dietary fiber [16, 24]. The diversity of intestinal microflora plays an important role in the production of SCFAs. Most SCFAs are produced in cecum and proximal colon, *Bacteroidetes* and *Firmicutes* are the two dominant bacteria producing SCFAs [30]. Acetate, butyrate, and propionate are the most abundant in intestinal SCFAs, and butyric acid can be produced by *Firmicutes* and *Bacteroidetes* [31]. As shown in Figs. 3 and 6, the content of SCFAs in feces all increased with the increase of the abundance of *Firmicutes* and *Bacteroidetes* from 6-month-old. SCFAs are considered to be an endogenous protective substance, which can decrease the gut lumen PH, inhibit the growth of pathogens, and regulate immune cell function through SCFA receptor on cell surface to maintain intestinal microecology stability [16]. In particular, butyric acid can enhance the intestinal epithelial barrier and modulate the gut immunol function [32]. As our results shown, the concentrations of three major SCFAs in stools, acetate (AA), propionate (PA), and butyrate (BA), were significantly higher than isobutyrate (IBA) and isovalerate (IVA), and the concentrations of fecal SCFAs kept in a lower lever during the neonatal period (0–28 d after birth), then increased at 6 months old and gradually tended to be stable after 2 years old (Fig. 6). The change of SCFAs was observed to be accompanied with the development of gut flora from birth to 6 years old.

Another important function of human intestinal microorganisms is to decompose primary bile acids (PBAs) through the liver-gut circulation of bile acids, and then convert them into secondary bile acids (SBAs) which are more meaningful for human health [15]. The microbial metabolism of bile acids increases the diversity of bile acids and the hydrophobicity of bile acid pools, which is conducive to the fecal excretion of bile acids. The mass of SBAs produced by intestinal flora is about 5% of the total bile acids [33]. Most bile acids (90%) are reabsorbed at the end of the ileum via the apical membrane Na dependent bile salt transporter (ASBT). The PBAs that are not reabsorbed by ASBT enter the colon and are metabolized into SBAs by 7-dehydroxylation under the action of *Firmicutes* in the intestinal flora [15]. In addition, *Firmicutes* and *Bacteroidetes* are also involved in the microbial transformation of bile acids, which produce oxygen- (or keto-) bile acids under the action of these bacteria containing hydroxysteroid dehydrogenase (HSDHs) [15, 33]. From the results of this study, the fecal contents of bile acids, especially SBAs, increased from 6-month-old, which accompanied with the increasing of the abundances of *Firmicutes* and *Bacteroidetes* in intestinal flora after neonate period (seen Figs. 3 and 5). Bile acids have endocrine functions and participate in the regulation of host metabolism, especially fat metabolism [15]. Therefore, bile acid composition is of great significance to human health. The crucial relationships between microbial bile acid metabolism and inflammatory bowel disease (IBD, i.e., ulcerative colitis and Crohn’s Disease), liver cirrhosis, liver cancer, irritable bowel syndrome, jejunal syndrome, obesity and other diseases have been confirmed [15, 34]. As seen in Fig. 5, the concentrations of fecal PBAs had no significant change from birth to 6 years old, while the fecal SBAs kept in a lower level during neonatal period and began to increase from 6 months old. Because the production of SBAs was more depended on the composition of gut microbiota, the content of fecal SBAs in infants was also according to the development of intestinal flora. At the same time, the time when SBAs content changed significantly and the change trend with age was consistent with the change of SCFAs content (Figs. 5 and 6). About 6 months after birth was a key time point for the increase of SBAs and SCFAs.

However, this study also has several limitations. First and most important, since this study is not a prospective cohort study, the effects of possible confounding factors such as mode of production (natural delivery, cesarean delivery), feeding mode (breastfeeding, milk powder feeding or mixed feeding), dietary conditions (dietary fiber, dietary culture or food habits) on the development of children’s intestinal flora were not considered, the results of this study may have conclusion deviation. Second, because the participants involved in the study were not the same group of children who were followed up from birth to preschool, but nine groups of participants from different ages, the research results could not directly reflect the dynamic changes of intestinal flora development in the growth process from newborns to preschool children, but only reflected the differences in the composition of intestinal flora of subjects at different ages. In order to get more accurate conclusions, in the follow-up study, the subjects need to be followed up longitudinally to obtain continuous data, and then the dynamic development and changes of intestinal flora in the growth and development of preschool children are confirmed through prospective longitudinal study. As well, in the process of the research, factors such as delivery mode, feeding mode, dietary structure and changes in health status should be included in the scope of research, so as to analyze whether the development and changes of intestinal flora are affected by these factors.

## Conclusion

In conclusion, the colonization and development of intestinal microflora in children mainly occurred in the first 3 years of life. The age of 6 months was a critical period of children development. At this stage, the abundance and function of intestinal flora began to increase and improve, which significantly promoted the metabolism of SCFAs and BAs.

## Supplementary Information

**Additional file 1: Figure S1.** The overview of the study design.

## Data Availability

The datasets used and/or analyzed during the current study are available from the corresponding author upon reasonable request.

## Abbreviations

SCFAs: Short chain fatty acids
AA: Acetic acid
PA: Propionate
BA: Butyrate
IBA: Isobutyric acid
IVA: Isovaleric acid
BAs: Bile acids
PBAs: Primary bile acids
CA: Cholic acid
CDCA: Chenodeoxycholic acid
SBAs: Secondary bile acids
LCA: Lithocholic acid
DCA: Deoxycholic acid
UDCA: Ursodeoxycholic acid
LC-MS: Liquid chromatography-mass spectrometry
GS: Gas Chromatography
HSDHs: Hydroxysteroid dehydrogenase
IBD: Inflammatory bowel disease
