# Supplementary figures and images for: Development of gut microbiota along with its metabolites of preschool children
Source: BMC Pediatr. 2022 Jan 6;22:25. doi: 10.1186/s12887-021-03099-9 (PMC8734072; doi:10.1186/s12887-021-03099-9)

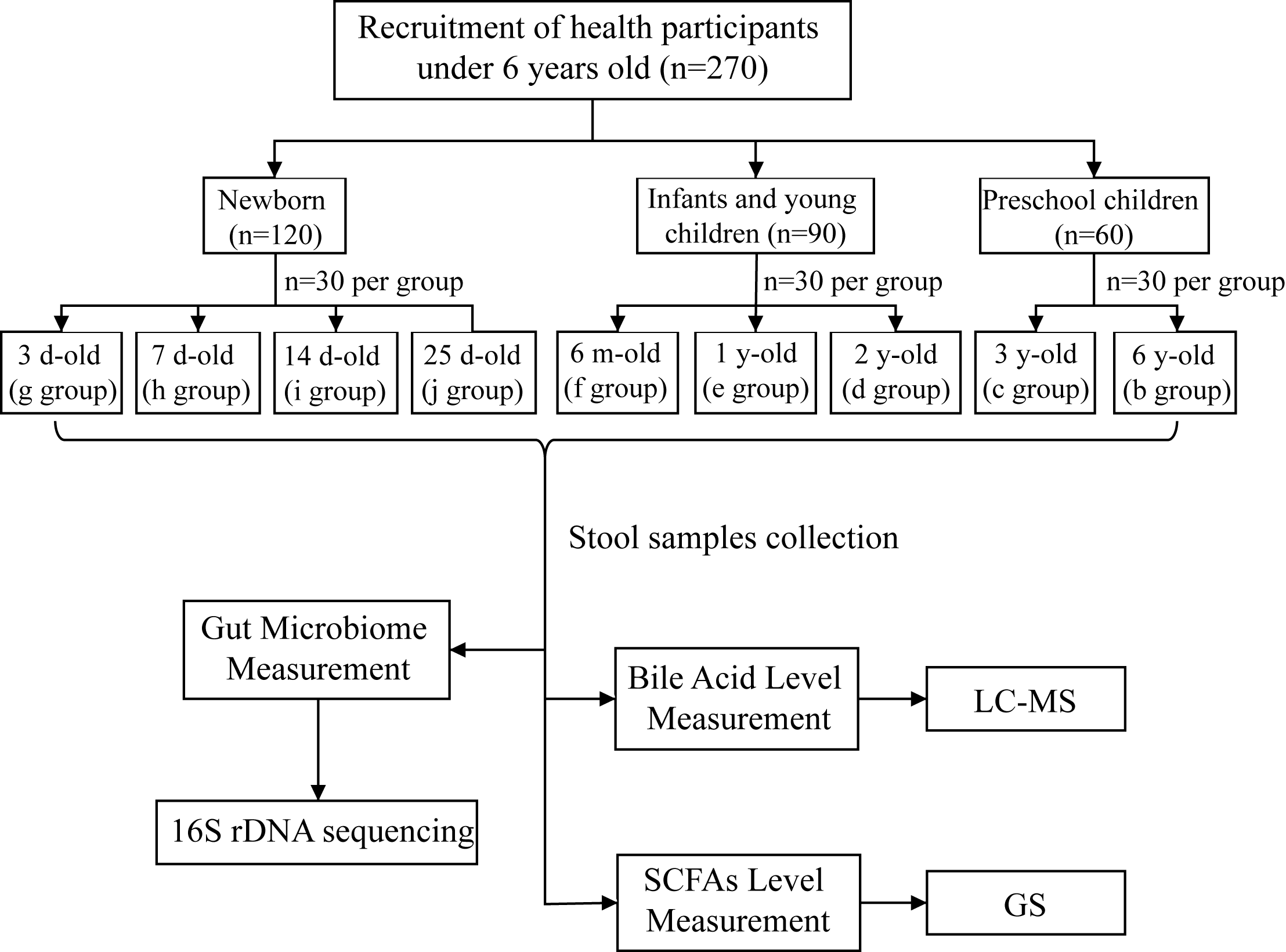

Supplement: Supplementary file 1 — Additional file 1: Figure S1. The overview of the study design. [file 12887_2021_3099_MOESM1_ESM.tif]
